# Are there statistical links between the direction of European weather systems and ENSO, the solar cycle or stratospheric aerosols?

**DOI:** 10.1098/rsos.150320

**Published:** 2016-02-17

**Authors:** Benjamin A. Laken, Frode Stordal

**Affiliations:** Section for Meteorology and Oceanography, Department of Geosciences, University of Oslo, Oslo, Norway

**Keywords:** European weather, ENSO, NAO, solar cycle, stratospheric aerosols

## Abstract

The Hess Brezowsky Großwetterlagen (HBGWL) European weather classification system, accumulated over a long period (more than 130 years), provides a rare opportunity to examine the impact of various factors on regional atmospheric flow. We have used these data to examine changes in the frequency (days/month) of given weather systems direction (WSD) during peak phases in the North Atlantic Oscillation (NAO), El Niño Southern Oscillation (ENSO), solar cycle (SC) and peaks in stratospheric aerosol optical depth (AOD) with superposed epoch analysis and Monte Carlo significance testing. We found highly significant responses to the NAO consistent with expectations: this signal confirmed the utility of the HBGWL data for this type of analysis and provided a benchmark of a clear response. WSD changes associated with ENSO, SC and AOD were generally within the ranges expected from random samples. When seasonal restrictions were added the results were similar, however, we found one clearly significant result: an increase in southerly flow of 2.6±0.8 days/month (*p*=1.9×10^−4^) during boreal summertime in association with El Niño. This result supports the existence of a robust teleconnection between the ENSO and European weather.

## 1. Introduction

To test if the atmospheric flow over the European region shows statistically significant changes in direction in response to peak phases of El Niño Southern Oscillation (ENSO), the solar cycle (SC) or increases in stratospheric aerosol optical depth (AOD), we have examined records of subjectively classified synoptic-scale weather systems. These data were recorded in Germany at a daily resolution over approximately 130 years by the Großwetterlagen classification method. This classification system was originally introduced by Baur *et al.* [1] and later improved and updated by Hess & Brezowsky [2]. We shall refer to these data as HBGWL throughout this work. Our analysis includes an examination of both monthly deseasonalized data and deseasonalized data grouped into four seasons.

We have also included a comparison of these data to peak phases of the North Atlantic Oscillation (NAO). The NAO should be highly associated with weather across Europe, and thus should provide us with a sanity check, ensuring that the subjective HBGWL data are producing a sensible and physically consistent result (e.g. as reported by [3]). Additionally, a NAO–HBGWL comparison should also act as benchmark of what a significant signal should look like in our analysis.

HBGWL data are broadly classified into cyclonic or anti-cyclonic types determined by the maximum and minimum daily temperature, pressure and precipitation from meteorological stations at Potsdam, Hamburg, Karlsruhe and Munich [4]. At the lowest level, the HBGWL are divided into 29 weather types (plus one unclassified type), known as Großwetterlagen. These can be grouped together into super-types, referred to as Großwettertypen. In this work, we consider the direction (origin) associated with 26 of the 29 classified Großwetterlagen weather types (as grouped in table 5 of [5]). These directions correspond to the eight principal compass directions (cardinal and inter-cardinal directions). The relationship between the specific Großwetterlagen and origin direction is stated in table 1, drawn from translations made by James [5]. When discussing these data we will refer to the weather system direction (WSD), which relates to large-scale circulation patterns over the whole of Europe and the northeast Atlantic, with a primary focus on central Europe [5]. We also broadly refer to this as atmospheric flow. This metric should not be confused with the near-surface wind direction at a specific location in Europe.

**Table 1.** The association of 26 specific Hess Brewsky Großwetterlagen (HBGWL) synoptic types to the eight principal compass directions from James [5].

symbol	direction
NA NZ HNA HNZ HB TRM	north
NEA NEZ	northeast
HFA HFZ HNFA HNFZ	east
SEA SEZ	southeast
SA SZ TB TRW	south
SWA SWZ	southwest
WZ WS WA WW	west
NWA NWZ	northwest

Associations between solar activity and climate variability over the North Atlantic and European regions have been identified in palaeoclimate records (e.g. [6–11]). Although these studies provide motivation for continued investigation regarding solar–terrestrial forcings, they do not necessarily relate to global-scale climate change. The distinction between regional-scale climate variability and globally coherent climate responses in the context of a solar forcing has been discussed by Benestad [12] and also Lockwood [13].

Despite the indications from palaeoclimatic data, the short lengths of modern observational datasets in relation to SCs has limited the ability of statistical methods to discern solar impacts on climate [14,15]. The relatively short records have made it difficult to disambiguate potential solar signals from other factors operating over similar timescales, such as ENSO and volcanic activity, which may produce aliased signals [16–19]. The benefit of this present analysis is that as the HBGWL are a simple metric, they have been recorded over a long time period (more than 130 *years*), meaning these data may be well suited to identify and disambiguate potentially small amplitude but persistent signals. We are additionally motivated by the findings of prior studies that suggest these (and similar) weather data show associations to solar activity (e.g. [20–23]).

We have presented this work via an Open Science approach, making our code openly available as an IPython Notebook accessible from Laken [24], which also contains supplementary figures and tables supporting this analysis. Such practices serve to increase the transparency and credibility of scientific work by facilitating sharing, interoperability, reproducibility and rapid modification. For a recent discussion of the importance of such practices to Climate Science, see Benestad *et al.* [25]. For further details on the IPython and Figshare tools see Singh [26] and Shen *et al.* [27].

## 2. Data and methods

### 2.1 HBGWL data

**Figure 1.**
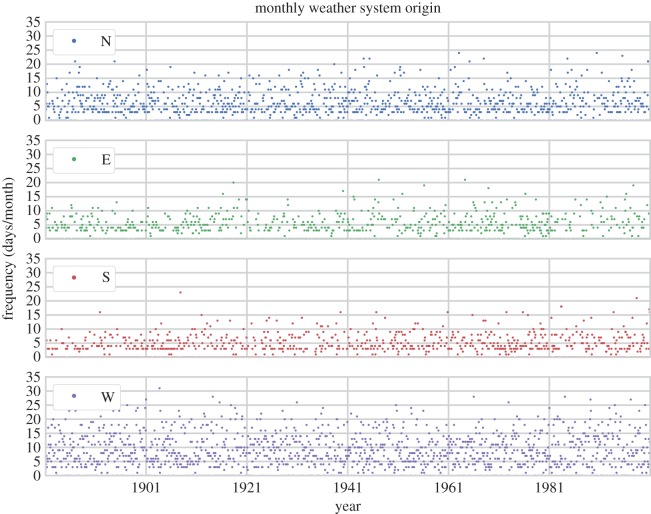
Frequency (days/month) of weather systems coming from cardinal compass directions, from the European (HBGWL) synoptic type data, from January 1881 to December 2000.

The HBGWL data were obtained from Gerstengarbe *et al.* [4]. In their original format they are a list of daily Großwetterlagen classification codes between 01/01/1801 and 31/12/2000. We have used the direction associated with each Großwetterlagen from James [5] to convert these data into monthly frequencies (days/month) over the eight principal (cardinal and inter-cardinal) compass directions, as shown in figure 1 for the cardinal directions. We will briefly consider the properties of these data prior to our analysis.

**Figure 2.**
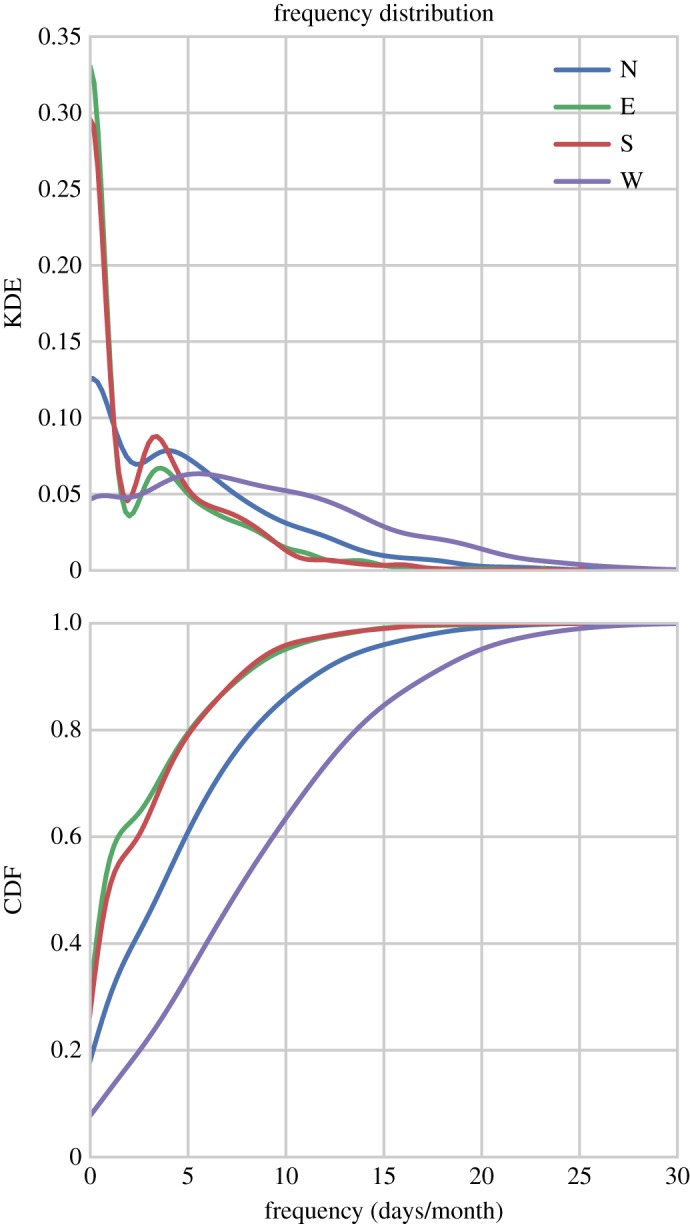
Kernel density estimates (KDEs) and cumulative density functions (CDFs) of the frequency (days/month) of weather systems coming from the cardinal compass directions.

The frequency distributions of the WSD are shown in figure 2 for cardinal compass directions using Kernel density estimates (KDEs) and cumulative density functions (CDFs). Easterly, and southerly flows exhibit broadly similar density functions: both have distributions which show a double peak in low frequencies centred around 0 days/month and 3 days/month, rapidly declining thereafter, reaching asymptotic values by approximately 10 days/month. The northerly flows exhibit largely comparable behaviour, again showing a double peak at low values (although the peak at 0 days/month is reduced), and a broader secondary peak centred around 4 days/month. The northerly distribution also declines more slowly, reaching asymptotic values around 20 days/month. The westerly flows show the most distinct distribution, with a broad unimodal population, centred around 6 days/month which gradually declines until 25 days/month.

**Figure 3.**
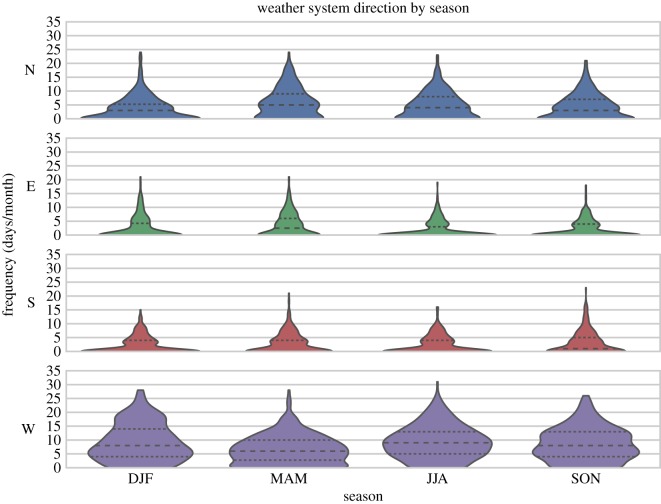
Violin plots showing the frequency (days/month) with which weather systems come from cardinal compass directions, grouped by season. Standard error of the mean values were on average 0.19 days/month and did not exceed 0.36 days/month. The violins, like box plots, show the first and third quartiles and median values on horizontal lines, in addition to kernel density estimations (KDEs) reflected around the centre of the categorical sample.

We have also distinguished these data by season, binning the monthly mean data into four categories (December–February, March–May, June–August and September–November). These are shown in figure 3 as violin plots, which present information in a manner similar to a box plots: they show the first and third quartile ranges with the median values on horizontal lines and extend to the maximum/minimum ranges of the data. However, unlike box plots, violin plots also include KDEs reflected around the centres of the seasonal categories with filled shading, giving the additional benefit of making a rapid comparison of the data distributions. The approach of presenting these data in categorical groups (as opposed to plotting continuous data across the compass directions) is important, as these data should not be thought of as continuous. Rather, they are indicative of subjectively defined regional atmospheric flow broadly grouped by direction. The violin plots show that westerly flow is dominant across all seasons, with an average of approximately 9 days/month except in Spring (March–May), during which time westerlies decrease to approximately 6 days/month, replaced by increased northerly and easterly flow. The distributions of the westerly data are either weakly bimodal or unimodal (depending on the season). This is consistent with the prevailing flow over Europe, which is dominated by westerly depressions originating from the North Atlantic storm track [28]. The violin plots also shows that the data are usually strongly bimodal for north, east and south flows, with predominant peaks around 0 days/month, and smaller secondary peaks around 5 days/month.

**Figure 4.**
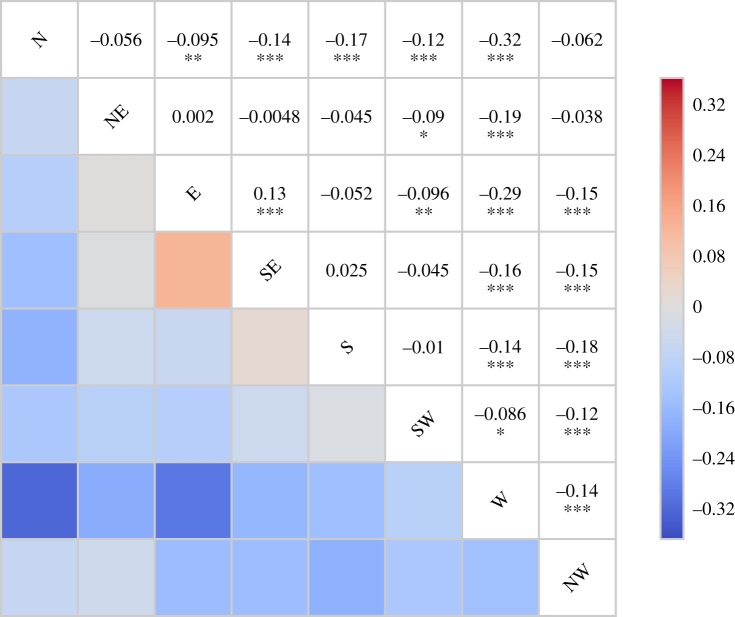
Correlation matrix of Pearson’s *r*-values showing the associations between the frequency (days/month) of various weather system directions. The strongest (anti-correlated) relationship is between western and northern flows. The statistical significance from permutation tests is indicated by asterisks. The correlation pattern and statistical significance remain when the data are deseasonalized.

Figure 4 shows a Pearson’s *r* correlation matrix of the frequency (days/month) of WSD from the principle compass directions. Almost all *r*-values are negative, with the most statistically significant correlations of −0.33 and −0.29 occurring between northerly to westerly and easterly to westerly directions, respectively. These data show that month-to-month changes in westerly flow often coincide with significant anti-phase changes in northerly and easterly flow. Only one direction pair showed a statistically significant positive correlation (of *r*=0.13), easterly to southeasterly. The lack of other positive relationships between adjacent compass directions may suggest that these data are biased towards the cardinal compass directions: i.e. the fact that more positive associations between closely related flow directions may indicate a bias towards selecting weather-types corresponding to cardinal directions.

Before any analysis of changes in the direction of weather systems associated with given forcings, seasonal variability is removed from these data. This is achieved by subtracting monthly climatological means from the dataset. All resulting data are described as an anomaly, denoted by *δ*. We note that following deseasonalization, these frequency data continue to show significant correlations between directions as described in figure 4.

### 2.2 NAO, ENSO, SC and AOD data

**Figure 5.**
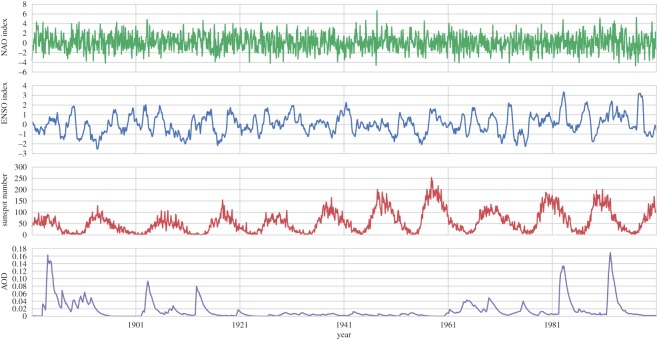
Time series of monthly averaged North Atlantic Oscillation (NAO) index, extended multivariate El Niño Southern Oscillation index, Wolf Sunspot number and Northern Hemisphere aerosol optical depth (AOD) from January 1881 to December 2000.

Monthly data for the NAO index, ENSO index, the SC from the Wolf Sunspot number and Northern Hemisphere stratospheric AOD are presented over the period 1881–2000, corresponding to the HBGWL data in figure 5. We broadly refer to these datasets as forcings or factors. We will now briefly describe the source and meaning of these data.

The NAO index is a measure of the mean atmospheric pressure gradient between the Azores High and the Icelandic Low, and provides an indication of the strength and position of the North Atlantic storm track and the strength of its associated westerly winds [28,29]. It is one of the large-scale modes of natural climate variability in the Northern Hemisphere, and is significant to the climate of the North Atlantic and Northern Europe, particularly in Boreal wintertime [30–32].

A positive NAO index in wintertime relates to a recurrent NAO configuration, characterized by a strong storm track, with a northeastward orientation driving depressions into northwestern Europe, while a negative NAO index can relate to weaker east–west oriented storm track, taking depressions into the Mediterranean region [28]. We have used the NAO index of Jones *et al.* [33], based on pressure observations from Gibraltar and southwest Iceland, extending back to 1823 (an updated version of which is maintained by Timothy Osborne at http://www.cru.uea.ac.uk/~timo/datapages/naoi.htm).

ENSO is the most important ocean–atmosphere interaction globally, driving climate variability over inter-annual timescales. We have used the extended multivariate ENSO index, described in Wolter & Timlin [34], with further details in Wolter & Timlin [35,36]. This index is based on reconstructions of sea-level pressure and sea surface temperature from the Hadley Centre back to 1871. Monthly values are computed separately from 12 sliding bi-monthly groups (e.g. December/January, January/February, etc.) each year, using the gridded values of the first unrotated principal component from the pressure and temperature fields (further details in [34]). Negative ENSO index values relate to the La Niña phase, while positive ENSO index values correspond to El Niño.

Teleconnections between ENSO and the climate of disparate parts of the globe, including Europe, have long been suggested (e.g. [37,38]). This includes claims based on evidence drawn from the HBGWL data [39]. Links have also been proposed between ENSO and the NAO, with observations suggesting negative NAO phases are increased in wintertime during El Niño, whereas positive NAO phases are increased during wintertime in association with La Niña events [40,41]. Mechanisms include the impact of anomalous sea surface temperatures on generating large-scale convection and overturning in the tropical atmosphere, and corresponding subsidence in the sub-tropical descending branch of the Hadley circulation, producing planetary-scale Rossby waves [42].

Solar activity is represented here by the Wolf Sunspot number, also known as the International or Zürich Sunspot number, developed by Rudolf Wolf in the nineteenth century [43]. These data are based on daily counts of sunspot groups and individual spots from direct observations of the Sun, corrected for differences in observers, and have been maintained approximately back to 1817 [44–46]. We make no effort here to distinguish the individual parameters that vary with solar activity, such as energetic solar particles, galactic cosmic rays, total solar irradiance, ultraviolet spectral irradiance and the Earth’s geomagnetic activity. For our purposes, the specific theoretical forcing is not of concern (for a summary of the proposed mechanisms, see [47]), rather, this work examines the statistical evidence of a regional-scale dynamic response to the SC in HBGWL data.

Finally, we also examined the monthly mean stratospheric AOD at 550 nm for the Northern Hemisphere. These data are described in Sato *et al.* [48] (updated data obtained from http://data.giss.nasa.gov/modelforce/strataer/). AOD data are intriguing in the context of this study for several reasons: major volcanic eruptions have been proposed as a significant forcing affecting the NAO (e.g. [49,50]), in which case we may expect to see NAO-like responses in the AOD samples of our study. Additionally, it has been shown that the last major volcanic eruptions (El Chichón in April 1982 and Mount Pinatubo in June 1991) coincided with solar maximum phases, and consequently may appear to alias the SC [19]. As our time series extends over 11 SCs, we should be able to disambiguate between volcanic and solar signals if they are present in the data. Therefore, a strong NAO-like response in the AOD composite lacking in the SC composite will lend support to the idea that the importance of solar forcings in the recent past has been over-estimated owing to the chance aliasing of a volcanic forcing signal [51].

### 2.3 Composite sample selection

To examine how the frequency of weather systems coming from different directions changes in association with each of our selected forcings, we have used an epoch super-positional (*aka* composite) sample technique [52], with the composites centred on the peaks of each forcing. Detailed explanations regarding the effective use of composites in similar climate investigations are given in Laken & Čalogović [53]. Our composites have been designed to give a good compromise between isolating a strong forcing while also making the resulting samples as comparable with each other as possible.

We identified the dates of our composites in several different ways depending on the forcing dataset: the solar maximum and minimum composites are simply based on the historical dates of the peak phases of the 11 year SC determined by NOAA. As there have been 11 SCs over the co-temporal data period our composites will each have a sample size (*n*) of 11 events. Consequently, in order for all samples to be directly comparable, we will maintain the *n*=11 sample size throughout the composites of the other forcing parameters also.

We identified peak NAO and ENSO phases by ranking these data, and then removing any dates which reoccurred within a ±365 day period of a stronger date (so that multiple composite dates were not drawn from the same year). The strongest 11 events for positive and negative phases were selected from the remaining list and composited. We use a slightly modified procedure to generate the AOD composite, extending the exclusion period to ±5 years to approximately draw the events from distinct volcanic episodes, so as to isolate a well-defined peak in AOD.

We reiterate that this procedure creates seven distinct composite samples, each based on a different list of 11 dates. One for each phase of the NAO, ENSO and SC and one for peaks in AOD. These samples will hereafter be referred to as positive NAO, negative NAO, El Niño, La Niña, solar maximum, solar minimum and peak AOD.

**Figure 6.**
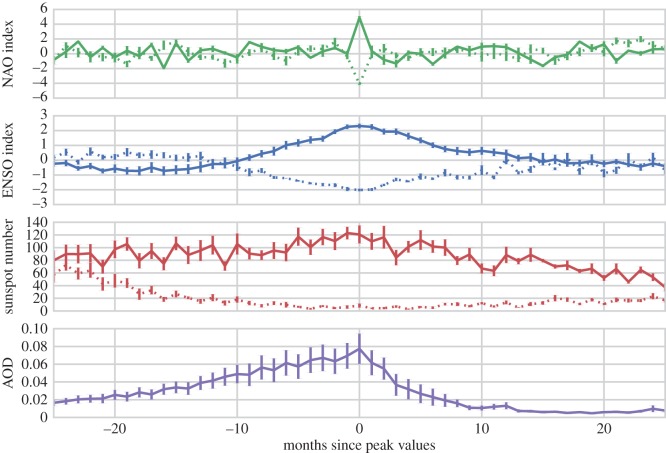
Superposed epoch (composite) samples for extreme phases of the various forcings shown in figure 5. For NAO and ENSO indices, the values are ranked, and the *n*=11 most extreme months from Jan 1801 to Dec 2000 (non-overlapping within a period of ±365 days) are selected as samples. The composite samples representing the extreme phases of the 11 year SC are identified from the months of solar maximum and solar minimum (*n*=11). Uncertainty ranges show ±1 s.e.m.

The factors isolated by each of the seven composite samples are presented in figure 6 over a ±24 month period, with solid lines corresponding to positive sample phases (positive NAO, El Niño, solar maximum and increased AOD), and broken lines corresponding to samples of negative phases (negative NAO, La Niña and solar minimum). Clear signals centred on the key composite months (lag 0) can be seen for each forcing parameter beyond the uncertainty of the data. At lag 0, the NAO index fluctuates by approximately ±4, this change is immediate, with values at epoch ±2 showing no clear disturbance. Whereas for ENSO the disturbances occur over a period of around ±10 months, peaking at values of ±2. The solar samples are different, with solar maximum and minimum appearing as different states rather than a peak centred around lag 0. This is owing to the relatively long transition between the phases of the SC. Consequently, the sunspot numbers around epoch ±24 in figure 6 have not returned to an undisturbed state as they more or less did for NAO and ENSO. Rather, they are transitioning towards the opposite solar phase, and thus the maximum and minimum composite samples become more similar to each other (yet maintain a non-random structure) around epoch ±24. Finally, the AOD composite shows a clear peak at lag 0 of approximately 0.8, however, it has a relatively long and asymmetrical lag: the signal begins prior to month −24 building slowly, the values return to undisturbed levels within approximately 12 months of their peak disturbance.

As a caveat we note that while it may be possible to further restrict or otherwise improve our composite methods to isolate a stronger signal from a specific forcing parameter, we have chosen an approach that maximizes our ability to compare the results across all forcings. As such, we have designed the composites to be drawn from distinct years (owing to autocorrelation in weather), and from distinct forcing events (e.g. different volcanic eruptions and different SCs) so that lags can be taken into account without interference from forcings aliasing themselves. For our statistical tests (detailed in the following section) to properly relate to the composites we also needed to maintain a consistent sample size. We acknowledge that this was a sub-optimal approach in some instances, for example, in the case of the AOD composite, wherein relatively weak eruptions were unavoidably included in the composite sample, potentially diminishing signal. However, significant signals are successfully identified for each forcing (as we have shown in figure 6), and, as we will demonstrate, these compromises provide us with the opportunity to readily compare the effect of the forcings. Furthermore, we note that users may optimize these experiments in any manner they wish via the Open Source code in Laken [54].

### 2.4 Monte Carlo significance testing

Composite analyses of geophysical data can be difficult to evaluate as they tend to be neither random nor sequentially independent, violating assumptions of many standard statistical procedures [55]. To address this issue, we have employed Monte Carlo (MC) significance testing, which involves drawing large numbers of composites from a dataset using randomized key dates. These composites are used to generate a distribution corresponding to the null hypothesis, against which samples may be evaluated. That is, these samples represent the possible range of values that you may expect to observe from a given dataset, for a given composite size, in the absence of a forcing. This is an approach that has long been used in solar–terrestrial analyses (e.g. [56,57]). For a detailed explanation of MC testing and its application to geophysical data relating to the field of solar–climate studies, see Laken & Čalogović [53].

**Figure 7.**
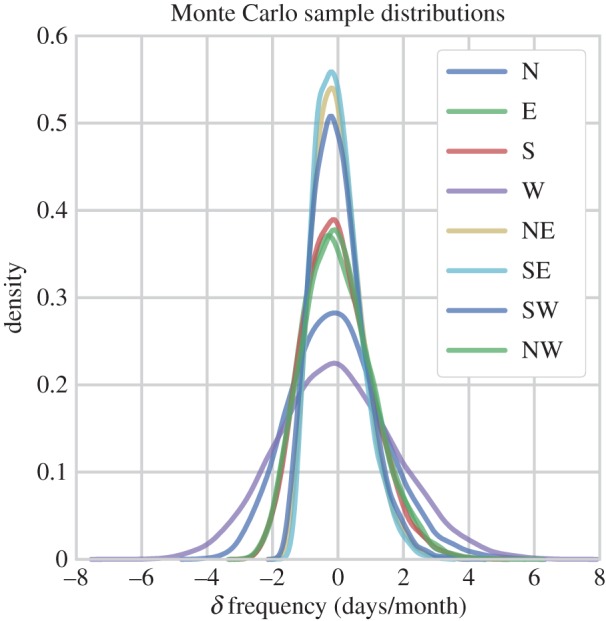
Kernel density estimates (KDEs) of deseasonalized (*δ*) WSD frequency (days/month) composites (*n*=11). The distributions were generated by MC sampling, in each instance creating 10 000 random composites. The distributions essentially show the range of values covered by the null hypothesis. That is, the variation in *δ* frequency with which weather systems may be expected from each direction in the absence of a forcing agent. KDEs estimated using the Seaborn.kdeplot Open Source software.

The specific MC-procedure used here involves randomly compositing the WSD *δ* frequency from samples of *n*=11 for each of the compass directions. Essentially, we compare our forcing sample composites to the MC distributions to estimate the probability (*p*) with which those values may have occurred by chance. To clearly explain the specific methods by which *p*-values are identified in our investigation we must first briefly describe the properties of the MC-generated populations themselves: to illustrate, we present figure 7, which shows KDEs from each of the compass directions, calculated from 10 000 random samples (using the Open Source software Seaborn.kdeplot). The distributions are approximately normally distributed around zero as a result of using the *δ* data. The more peaked and narrow distributions belong to directions with the least variance (northeast, southeast, northwest), with a peak density of approximately 0.55, covering a range of ±2 days. Whereas, the broadest distribution is the westerly direction, with a peak density of approximately 0.22 and a slightly positive skew, covering a range of values between −7 and 8 days/month. We note that the distributions shown in figure 7 are created from *δ* monthly data, and thus may only be used to identify the statistics of our forcing composites for the *δ* monthly data. That is, in order to test the *p*-value of the DJF, or JJA data we must generate new MC distributions.

In our analysis, we use the MC distributions to provide two different methods of estimating probability: (i) a *simple visual method*, whereby crude confidence intervals from percentiles of the MC-populations are projected onto our plots for a qualitative assessment; and (ii), a *precise estimate* of the *p*-value of the composite mean and uncertainty range based on Gaussian KDE fits to the MC-generated populations, made using the Scipy.stats.gaussian_kde function of the SciPy library. A description of how these tests are used is given in the following section.

## 3. Results

### 3.1 How to interpret the composites and notes on significance

We present our composite results in a consistent fashion: for each cardinal direction (ordered in the *x*-axis dimension) the WSD mean and standard error of the mean (s.e.m.) *δ* frequency are over-plotted on the range of null-cases indicated by the MC samples. The null-cases are indicated as a band of grey shading, where the central portion of the band’s shading covers the 5th–95th percentile range of the MC population (this approx. encompasses values of *p*>0.10), the middle band extends from the 2.5th–97.5th percentile interval (indicating the *p*=0.05 significance level), and the outer band extends to the 0.5th–99.5th percentile interval (indicating the *p*=0.01 significance level). (This is our *simple visual method* of indicating statistical significance, as mentioned in the previous section.) For each composite experiment our results are split across two panels to avoid overcrowding the plot: i.e. there are different sets of plots for the monthly, DJF, and JJA experiments. We note that we do not present the MAM or SON figures directly as they lacked interesting results.

We have maintained a consistent aesthetic throughout, so that the triangle symbols on the plots indicate composite samples where the forcing was in a positive phase (i.e. positive NAO, El Niño, solar maximum), while circle symbols indicate a negative phase (negative NAO, La Niña, solar minimum). Likewise, colours are consistent across plots, with green indicating the NAO, blue for ENSO, red for the SC and purple for the AOD.

The precise values of each composite are also listed in companion tables, including a *precise estimation* of the *p*-value for the mean, and upper/lower mean uncertainty range (as mentioned in the previous section). There may be slight disagreement in *p* estimates between the grey shading and the precise values stated in the tables, as, depending on the size of the MC-generated populations, the *simple visual method* of estimating significance is likely to be less accurate (particularly at the extreme tails of the distributions). For a discussion of the limitations and pitfalls of using MCs in this manner, see Laken & Čalogović [53].

Although we examined lags over a range of periods, we only present the lag 0 cases in this manuscript, as these were the most notable and relevant results. However, we note that figures at any lag may be generated by the supporting software [24].

### 3.2 Results from the monthly cases

**Figure 8.**
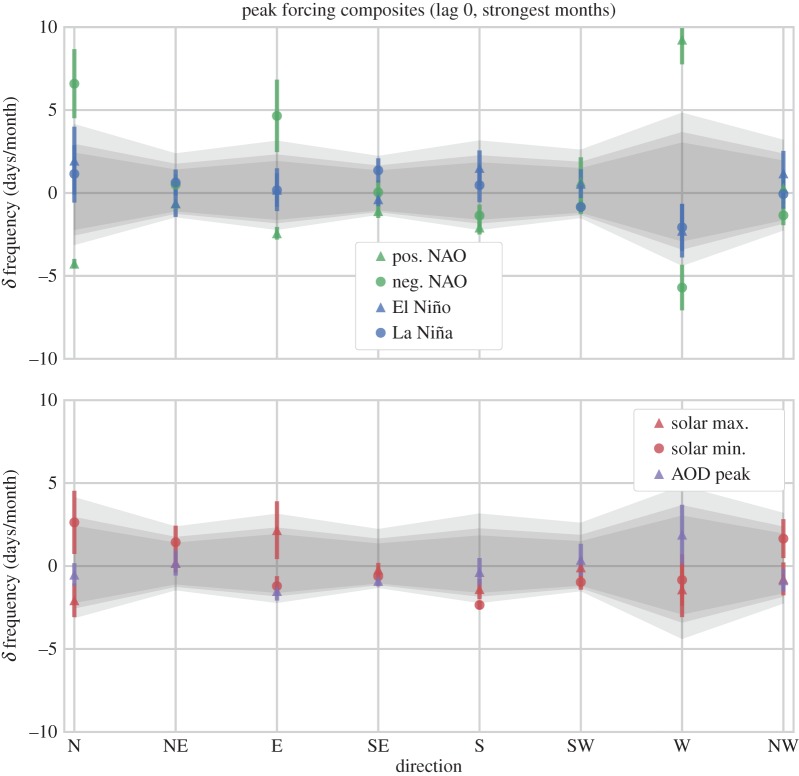
Deseasonalized (*δ*) frequency of weather systems coming from a given direction, during composites of extreme phases of the NAO, ENSO and SC during months of peak forcings. Values are displayed as simple means with an uncertainty of ±1 s.e.m. Grey shading indicates confidence intervals (estimated from the KDEs of figure 7), whereby the central shaded band indicates values of *p*>0.05 significance, and the lightest grey shading extends to the *p*=0.01 confidence level.

The results of the WSD *δ* frequency composites are shown in figure 8, with specific values listed in table 2. We recommend that you, the reader, interpret these figures in the following manner: first, you should note that the composites based on peak phases of the NAO sit well outside the range of null-values generated by the MC simulations. The statistical significance of these points is considerably high; e.g. there is an increase of 9.2±1.5 days/month for westerly weather systems in association with positive NAO conditions, with a mean significance estimated to be 1.98×10^−16^. Considering the mean uncertainty, the significance may be anywhere from 1.6×10^−5^ to 2.75×10^−35^. Anti-correlated anomalies of similar amplitude and significance can be observed between composites of positive and negative NAO phases. This validates the usefulness of the HBGWL data for our purposes, as they are responding in a consistent, significant, and expected fashion to the NAO. These responses should be treated as benchmarks of significant signals (i.e. when examining responses to the other forcing parameters, unambiguously clear signals should look comparable to these results).

**Table 2.** The *δ* frequency (days/month) of weather system direction, and estimates of probability for NAO, ENSO, solar and peak AOD composites at lag 0.

	N	NE	E	SE	S	SW	W	NW
pos. NAO	−4.27±0.29	−0.64±0.07	−2.43±0.38	−1.10±0.41	−2.08±0.42	0.69±1.46	9.24±1.494	0.30±1.01
*p*-val lower	7.29×10^−5^	0.42	8.79×10^−4^	0.01	0.01	0.40	1.60×10^−5^	0.33
*p*-val mean	4.20×10^−4^	0.46	0.01	0.19	0.04	0.28	1.98×10^−16^	0.32
*p*-val upper	0.00	0.49	0.05	0.45	0.12	0.03	2.75×10^−39^	0.16
neg. NAO	6.59±2.08	0.50±0.61	4.65±2.18	0.04±0.75	−1.36±0.65	−0.84±0.43	−5.71±1.37	−1.35±0.59
*p*-val lower	0.00	0.53	0.03	0.44	0.05	0.16	2.95×10^−8^	0.07
*p*-val mean	1.55×10^−4^	0.33	5.78×10^−4^	0.51	0.19	0.37	5.08×10^−4^	0.21
*p*-val upper	7.49×10^−28^	0.16	2.98×10^−19^	0.25	0.34	0.51	0.01	0.33
El Niño	1.95±2.05	−0.62±0.82	0.20±1.29	−0.38±0.42	1.51±1.06	0.55±0.87	−2.28±1.61	1.18±1.35
*p*-val lower	0.28	0.06	0.27	0.39	0.31	0.51	0.02	0.36
*p*-val mean	0.10	0.47	0.33	0.59	0.12	0.32	0.10	0.18
*p*-val upper	0.01	0.46	0.13	0.51	0.03	0.10	0.21	0.03
La Niña	1.15±1.73	0.63±0.78	0.17±1.01	1.36±0.73	0.47±1.02	−0.84±0.31	−2.07±1.42	−0.07±0.88
*p*-val lower	0.27	0.53	0.32	0.30	0.36	0.21	0.03	0.29
*p*-val mean	0.18	0.30	0.34	0.10	0.31	0.36	0.12	0.36
*p*-val upper	0.04	0.10	0.18	0.02	0.12	0.48	0.21	0.25
solar max.	−2.06±1.01	0.20±0.60	2.16±1.74	−0.21±0.40	−1.40±0.60	−0.08±0.59	−1.41±1.67	−0.77±0.99
*p*-val lower	0.02	0.52	0.30	0.50	0.05	0.44	0.05	0.10
*p*-val mean	0.11	0.46	0.05	0.58	0.18	0.48	0.17	0.32
*p*-val upper	0.23	0.25	0.00	0.47	0.32	0.34	0.21	0.33
solar min.	2.63±1.90	1.43±1.00	−1.21±0.59	−0.61±0.28	−2.35±0.25	−0.97±0.46	−0.84±1.55	1.65±1.17
*p*-val lower	0.23	0.36	0.10	0.34	0.01	0.10	0.09	0.30
*p*-val mean	0.05	0.10	0.25	0.50	0.02	0.30	0.21	0.11
*p*-val upper	0.00	0.01	0.35	0.59	0.04	0.49	0.20	0.02
high AOD	−0.52±0.69	0.18±0.75	−1.51±0.57	−0.90±0.32	−0.36±0.84	0.37±0.97	1.90±1.79	−0.86±0.68
*p*-val lower	0.22	0.48	0.05	0.11	0.23	0.47	0.21	0.15
*p*-val mean	0.28	0.47	0.16	0.33	0.37	0.39	0.12	0.31
*p*-val upper	0.27	0.21	0.30	0.52	0.31	0.11	0.03	0.36

Given this criteria, you will see that none of the other composite samples of ENSO, the SC or peak AOD in figure 8 show unambiguously significant changes. A case may be made for marginal significance, however, as there are several instances where composite values begin to cross into the tails of the null-distribution indicated by the grey-shaded intervals (our *simple visual method* of assessing significance). Composite means passing the second interval can be considered as significant at the *p*=0.05 level. Although, in many cases the uncertainty of these data is considerable, including a range of non-significant values. However, there are certainly samples which may be suggestive of relationships, such as the results from the solar minimum sample which shows a reduction of −2.35±0.25 days/month in weather systems coming from the southerly direction, and a mean *p*-value of 0.02, with an uncertainty covering a range of 0.01–0.04. Interestingly, this result closely resembles the corresponding values from the positive NAO composite, and also the values from the negative NAO composite, which are less significant.

When considering the interpretation of values such as this, we must consider the chance of false-positives dictated by the false discovery rate (FDR) [58]: with 56 composites samples presented in each set of figures, we may expect 2.8 samples to pass the *p*=0.05 significance threshold randomly. Thus, we recommend that marginally significant results such as these be interpreted cautiously, and not simply accepted as a reliable indication of a causal phenomena.

### 3.3 Composites with a seasonal restriction

Although the forcings we have examined were not associated with a clear signal in the HBGWL data, it is possible that adding a seasonal restriction will alter these results. For example, it has been argued that solar signals may become apparent in the wintertime period (DJF) over the European region, as the stratosphere becomes highly dynamically active in winter and can transfer solar signals from the stratosphere to the troposphere owing to the presence of the polar vortex; the so-called *polar route* solar influence [59]. Thus, we may expect to see significant HBGWL responses in the DJF solar composites absent in both composites of the other seasons and also the previously discussed composite of figure 8 (which was constructed irrespective of season). As such, our analysis has been repeated with a seasonal restriction.

We note that we have purposefully approached the investigation in this manner, systematically incrementing the restrictions placed on the data, as false-positives often arise as a result of excessive data restriction and overly-complex ad hoc hypotheses. These have often been associated with purported solar–climate responses which break down or even reverse sign over time [14]. Incrementing the complexity of our tests enables us to contextualise the results.

The procedure for obtaining our seasonal results was as follows: we averaged the WSD *δ* frequency (days/month) for each of the principle compass directions into four distinct time series, one for each seasonal period. We also constructed comparable seasonal mean time series relating to the forcing data. Each time series had one value per year, indicating monthly mean *δ* frequency averaged over the seasonal period. Uncertainty was estimated with the s.e.m., described in equation (3.1), where *n* is the number of months in the winter season, and *σ* is the sample standard deviation (as was used to estimate uncertainty in the previous composites).

We then applied a similar method to that used earlier to identify the composite dates, wherein the seasonal forcing data were ranked, and values recurring within a ±5-year period of stronger values were excluded from the list. (The exclusion period was required to prevent self-aliasing in a lagged analysis, and to obtain values from distinct SCs and volcanic episodes.) The strongest, *n*=11, seasons were then selected as the composite sample. To estimate mean error in the seasonal composites, we accumulated the s.e.m. uncertainty using equation (3.2).

3.1 $$s.e.m.=\frac{\sigma }{\sqrt{\left(n-1\right)}}$$

and

3.2 $$s.e.m.=\frac{1}{n}\sqrt{\sum_{i=1}^{n}{\left({s.e.m.}_{i}\right)}^{2}}.$$

**Figure 9.**
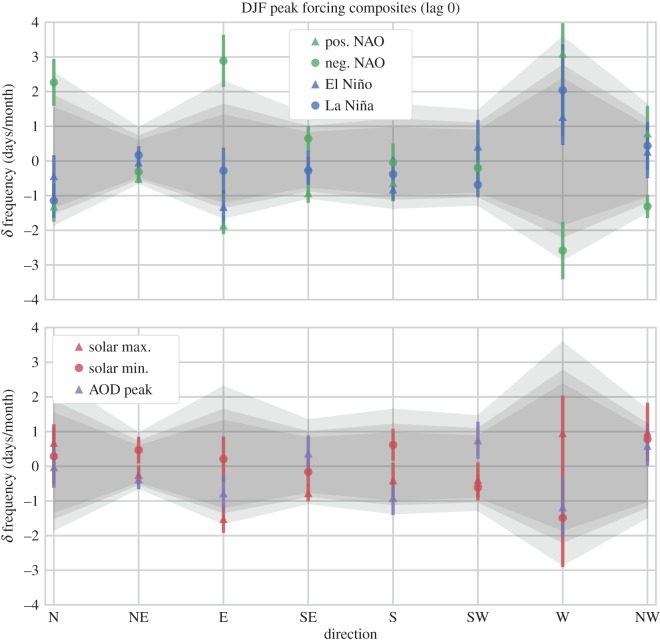
Deseasonalized (*δ*) frequency (days/month) of weather systems from a given direction, during composites of extreme phases of the NAO, ENSO, SC and AOD peaks for several epochs during winter (DJF) months. Values are displayed as simple means with an accumulated uncertainty (details in text). Grey shading indicates confidence intervals (estimated from the KDEs of 10 000 random DJF composites for each direction), whereby the central shaded band indicates values of *p*>0.05 significance, and the lightest grey shading extends to the *p*=0.01 confidence level.

**Figure 10.**
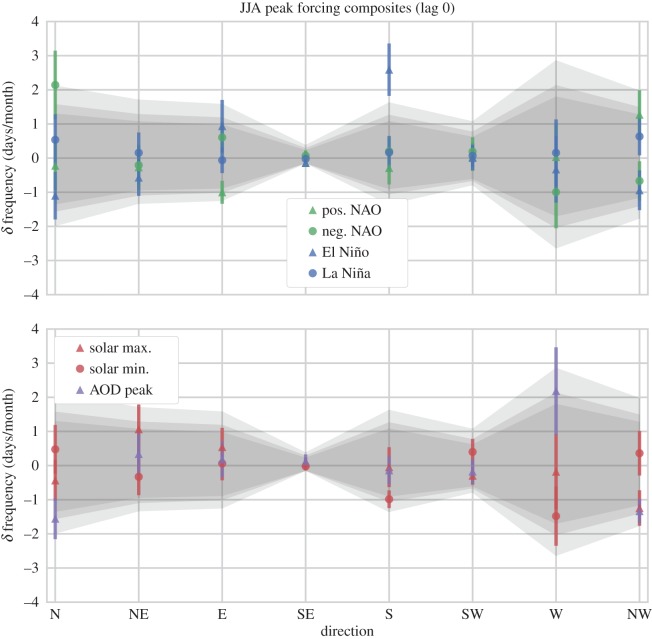
Same as for figure 9, except for JJA months. El Niño sample corresponds to a highly-significant increase in southerly flow (of approx. *p*= 1.9×10^−4^).

The resulting composite dates corresponded to peak phases of the NAO, ENSO, SC and peaks in stratospheric AOD that occurred over specific seasons. These results are shown in an identical format to the previous composites. As no robust significance was identified in the MAM or SON seasonal samples (except for further associations to the NAO), we have omitted these figures, leaving only those of the DJF wintertime composites (figure 9 and table 3) and JJA summertime composites (figure 10 and table 4). For further details including the specific composite dates identified, and MAM, SON figs and tables, see [24].

**Table 3.** The *δ* frequency (days/month) of weather system direction, and estimates of probability for NAO, ENSO, solar and peak AOD composites at lag 0 during the boreal winter (DJF) months.

	N	NE	E	SE	S	SW	W	NW
pos. NAO	−1.31±0.44	−0.51±0.13	−1.85±0.26	−0.93±0.28	−0.63±0.53	−0.10±0.55	3.10±0.98	0.80±0.79
*p*-val lower	0.04	0.11	0.00	0.02	0.12	0.45	0.10	0.61
*p*-val mean	0.16	0.34	0.01	0.13	0.50	0.72	0.03	0.25
*p*-val upper	0.35	0.70	0.05	0.40	0.67	0.45	0.00	0.03
neg. NAO	2.27±0.67	−0.31±0.21	2.89±0.75	0.65±0.36	−0.04±0.55	−0.21±0.39	−2.58±0.83	−1.31±0.34
*p*-val lower	0.09	0.30	0.02	0.62	0.54	0.48	0.00	0.01
*p*-val mean	0.02	0.92	0.00	0.31	0.65	0.72	0.03	0.07
*p*-val upper	0.01	1.27	1.90×10^−4^	0.09	0.36	0.62	0.10	0.22
El Niño	−0.43±0.60	−0.04±0.35	−1.33±0.50	−0.17±0.48	−0.82±0.32	0.42±0.77	1.27±0.81	0.27±0.77
*p*-val lower	0.28	0.67	0.02	0.40	0.13	0.64	0.29	0.54
*p*-val mean	0.46	1.26	0.12	0.81	0.36	0.47	0.21	0.53
*p*-val upper	0.42	0.66	0.37	0.60	0.58	0.07	0.10	0.15
La Niña	−1.15±0.49	0.17±0.26	−0.28±0.66	−0.28±0.41	−0.38±0.50	−0.69±0.36	2.04±1.32	0.44±0.69
*p*-val lower	0.07	1.27	0.32	0.35	0.31	0.15	0.27	0.64
*p*-val mean	0.23	0.94	0.51	0.76	0.63	0.42	0.11	0.45
*p*-val upper	0.42	0.44	0.39	0.73	0.59	0.65	0.02	0.12
solar max.	0.68±0.53	−0.25±0.27	−1.52±0.40	−0.77±0.24	−0.40±0.51	−0.39±0.50	0.96±1.08	0.99±0.85
*p*-val lower	0.42	0.32	0.01	0.08	0.29	0.23	0.31	0.57
*p*-val mean	0.28	1.11	0.06	0.25	0.63	0.60	0.24	0.17
*p*-val upper	0.16	1.21	0.23	0.53	0.60	0.65	0.11	0.01
solar min.	0.29±0.81	0.47±0.38	0.21±0.65	−0.16±0.40	0.62±0.46	−0.61±0.38	−1.49±1.42	0.78±0.45
*p*-val lower	0.46	1.11	0.50	0.49	0.58	0.18	0.02	0.50
*p*-val mean	0.40	0.40	0.45	0.81	0.30	0.48	0.14	0.26
*p*-val upper	0.18	0.08	0.22	0.65	0.11	0.71	0.31	0.09
high AOD	−0.03±0.59	−0.38±0.28	−0.78±0.51	0.37±0.52	−0.91±0.49	0.75±0.53	−1.18±0.84	0.59±0.58
*p*-val lower	0.43	0.09	0.14	0.81	0.04	0.61	0.07	0.61
*p*-val mean	0.44	0.72	0.39	0.54	0.29	0.25	0.18	0.37
*p*-val upper	0.31	1.27	0.51	0.15	0.62	0.05	0.29	0.10

**Table 4.** The *δ* frequency (days/month) of weather system direction, and estimates of probability for NAO, ENSO, solar and peak AOD composites at lag 0 during the boreal summer (JJA) months.

JJA	N	NE	E	SE	S	SW	W	NW
pos. NAO	−0.22±0.72	−0.27±0.66	−1.00±0.34	−0.14±0.02	−0.29±0.48	0.04±0.41	0.03±1.00	1.27±0.72
*p*-val lower	0.29	0.22	0.02	2.99	0.30	0.85	0.24	0.40
*p*-val mean	0.48	0.61	0.14	5.44	0.59	0.96	0.37	0.13
*p*-val upper	0.39	0.49	0.40	3.00	0.62	0.40	0.23	0.02
neg. NAO	2.14±1.00	−0.21±0.56	0.61±0.46	0.07±0.15	0.20±0.41	0.19±0.42	−1.00±1.06	−0.67±0.58
*p*-val lower	0.17	0.33	0.65	0.98	0.63	1.05	0.06	0.12
*p*-val mean	0.01	0.63	0.37	2.66	0.62	0.77	0.23	0.36
*p*-val upper	1.10×10^−4^	0.52	0.13	0.56	0.38	0.24	0.37	0.54
El Niño	−1.10±0.70	−0.57±0.53	0.94±0.76	−0.14±0.02	2.59±0.77	0.01±0.36	−0.33±0.98	−0.94±0.58
*p*-val lower	0.04	0.12	0.63	2.99	0.01	0.89	0.18	0.05
*p*-val mean	0.22	0.44	0.19	5.44	1.90×10^−4^	0.99	0.34	0.24
*p*-val upper	0.45	0.64	0.01	3.00	2.65×10^−13^	0.52	0.30	0.48
La Niña	0.54±0.75	0.16±0.59	−0.06±0.38	−0.02±0.12	0.17±0.48	0.07±0.33	0.16±0.98	0.63±0.55
*p*-val lower	0.48	0.52	0.56	5.29	0.58	1.04	0.27	0.54
*p*-val mean	0.37	0.59	0.69	4.62	0.63	0.93	0.37	0.36
*p*-val upper	0.13	0.29	0.56	1.86	0.35	0.48	0.22	0.16
solar max.	−0.43±0.60	1.07±0.72	0.54±0.56	0.07±0.15	−0.05±0.58	−0.30±0.26	−0.18±1.10	−1.24±0.52
*p*-val lower	0.24	0.52	0.69	1.15	0.40	0.48	0.18	0.02
*p*-val mean	0.45	0.16	0.41	2.66	0.67	0.99	0.36	0.12
*p*-val upper	0.47	0.02	0.12	0.57	0.42	1.02	0.25	0.34
solar min.	0.48±0.71	−0.33±0.54	0.06±0.49	−0.02±0.12	−0.99±0.26	0.40±0.38	−1.48±0.87	0.36±0.65
*p*-val lower	0.48	0.26	0.57	5.29	0.06	0.98	0.03	0.50
*p*-val mean	0.40	0.58	0.67	4.62	0.17	0.47	0.15	0.47
*p*-val upper	0.15	0.58	0.40	1.86	0.33	0.13	0.30	0.22
high AOD	−1.55±0.60	0.34±0.61	0.18±0.53	0.13±0.20	−0.14±0.41	−0.17±0.36	2.19±1.28	−1.33±0.36
*p*-val lower	0.01	0.60	0.61	2.71	0.47	0.54	0.25	0.03
*p*-val mean	0.08	0.52	0.63	1.36	0.65	1.05	0.05	0.09
*p*-val upper	0.28	0.21	0.32	0.17	0.59	0.78	0.00	0.22

Examining the boreal wintertime (DJF) composites, we see that during peak NAO phases the magnitude and significance of the HBGWL anomalies is decreased relative to the monthly analysis of figure 8. However, the pattern of change is similar, with positive (negative) anomalies being associated with increased (decreased) westerly flow and a corresponding decrease (increase) in both easterly and northerly flow. (We note that signals maintaining consistency across different and restricted samples is another good indication that the signals are reliable.) No other composite samples appear significant, and examining the *p*-values in table 3 confirms that the anomalies associated with ENSO, the SC and AOD peaks in wintertime failed to show robustly significant changes in WSD .

The reduced magnitude and significance of the observed HBGWL response to peak phases of the NAO was probably owing to the seasonal restriction procedure, which may have decreased the signal-to-noise ratio (SNR) by reducing the magnitude of the NAO anomalies isolated.

As expected, the boreal summertime (JJA) composites show the NAO phase has a diminished impact on European climate relative to winter months (figure 10): a result that agrees with conventional wisdom [60] and again supports the reliability of these data. Indeed, no unambiguously significant change in WSD frequency is observed in association with the positive or negative NAO samples, although composites of the negative NAO phase shows weather systems coming from the northern direction are associated with an increase of 2.14±1.0 days/month from seasonal averages, at a significance of *p*=0.01 with an uncertainty range of 1.1×10^−4^ to 0.17 (table 4). As before, the AOD and SC composites all fall within the range of expected values. Interestingly, however, a robustly significant result is observed for the El Niño composite: southerly weather systems are increased by 2.59±0.77 days/month, with a significance of *p*=1.9×10^−4^ and uncertainty range of 2.65×10^−13^ to 0.01.

**Figure 11.**
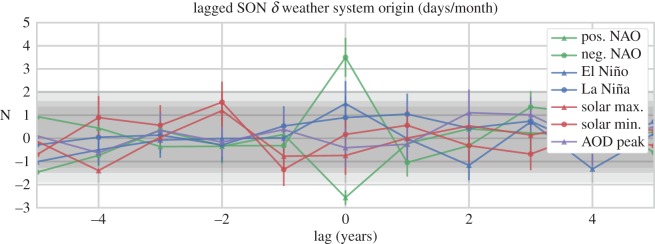
Deseasonalized (*δ*) frequency (days/month) of northerly weather systems, during lagged composites of extreme phases of the NAO, ENSO, SC and peaks in AOD, for several epochs during autumn (SON) months. Values are displayed as simple means with an accumulated uncertainty. Grey shading indicates confidence intervals estimated from a KDE of 10 000 random composites, wherein the central shading indicates values of *p*>0.05 significance, and the lightest grey shading extends to the *p*=0.01 confidence level. Highly significant (and opposing) changes in northerly flow are evident centred around peak phases of the NAO.

**Figure 12.**
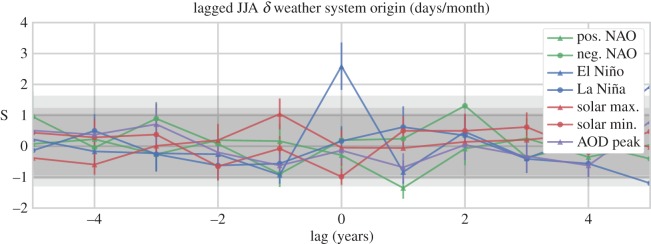
Same as figure 11 except for southerly weather types during boreal summer (JJA) months. A highly significant increase in southerly flow is evident centred around peak El Niño conditions.

To further test the strength of the boreal summertime El Niño result, we have examined the signal considering lags over a ±5 year period. Before discussing these results, however, we first present an example from the northerly data for the boreal autumn (SON) composites, for the purpose of seeing what a significant response looks like in the lagged data (figure 11). As with preceding figures, the grey shading indicates the confidence intervals at the *p*=0.1, *p*=0.05 and *p*=0.01 levels. However, in this case, the *x*-axis shows lag (in years) from the peak forcing, with all seven composite samples represented. The development of a clear, anti-correlated signal in both the positive and negative phase NAO composites, far outside of the range expected from the null cases, occurring directly on the year of peak forcing is evident, which then immediately abates. No other values clearly pass beyond the significance thresholds over the lag period. This signal serves as a good benchmark response. Compare this to the signal seen in the southerly boreal summertime data, shown in figure 12: a similar pattern emerges, no composite values pass the significance thresholds over the examined lag period beyond the expected FDR, except for those of El Niño, which emerges directly at the peak forcing. We interpret this result as strong evidence in support of a teleconnection between ENSO and European weather.

## 4. Discussion

This analysis has examined how the frequency with which European weather systems coming from different directions changes with peaks in a variety of parameters (which we have broadly referred to as forcings). We consider these data to be useful for testing notions of regional-scale circulation changes, in association with variables that are problematic to isolate over the relatively short timescales available with modern satellite-era instruments. However, the data have significant limitations, restricting our ability to reach definitive conclusions on all aspects of this research field. In particular, some relevant limitations concern:

(i) the insensitivity of the analysis to detect changes below broad shifts in the flow pattern categories;
(ii) the inability of these data to indicate second-order effects on weather systems, such as their possible invigoration or suppression (e.g. as suggested by [61]); and
(iii) the HBGWL data itself has significant uncertainty and limitations owing to the subjective nature of the classifications and the reliance on human observers over long periods; consequently, these data show long-term artefacts [62]. While the extent to which our analysis is affected by the imprecise nature of these data is unclear, Cahynová & Huth [62] noted that artefacts appear at monthly timescales in relation to unnaturally rapid changes between weather types at the ends of calendar months for HBGWL data, indicating that observer errors may certainly impact the reliability of our analysis.

There have been numerous reports of a regional atmospheric circulation response to solar activity from examinations of the past several decades of climate data, with many authors concluding that the data support a solar influence on European climate via a connection to the NAO (e.g. [20,63–69]). Shindell *et al.* [70] proposed that a solar-influenced change in the NAO during the Maunder minimum was the cause of the cold European winters from mid-1600s to the early 1700s. As stated by Lockwood *et al.* [22], the observations of Barriopedro *et al.* [71] that low-frequency (i.e. slow-moving) weather events correlate to solar activity is consistent with these ideas, as the position of such slow-moving or stationary blocking weather systems in the North Atlantic, and the frequency with which they occur are known to impact regional climate via the tropospheric jet stream [72]. Although the mechanism by which solar activity may influence the development of weather systems is unclear, Barriopedro *et al.* [71] conclude that if solar activity is able to alter the properties of blocking events, this could provide a link between solar activity and climate variability over Europe.

In relation to the HBGWL data, Huth *et al.* [20,65] have performed several analyses of the link between weather types and solar activity. Huth *et al.* [20] examined changes in the individual synoptic classifications during wintertime, dividing them into categorizations of high, moderate and low phases of solar activity (indicated by the solar radio flux at 10.7 cm). They conclude that at solar minimum there was a decrease in weather types with westerly flow and a corresponding increase of northerly and easterly flows, while periods of higher solar activity show increased northeasterly and northwesterly flow. These findings were in accord with their earlier work [65], which reported a strengthening of zonal flow during solar minimum (in this case using Wolf Sunspot data) with increased occurrences of low-frequency blocking weather events. It is changes in blocking events in particular which Barriopedro *et al.* [71] have argued are important for a solar link to regional climate variability.

Thus far, several mechanisms have been proposed to account for a solar influence on climate, primarily these are: bottom-up total solar irradiance related feedbacks [73], top-down solar spectral ultraviolet irradiance and stratospheric modification [74] such as via the previously mentioned *polar route* [59], and solar-wind modulated energetic particles and atmospheric ionization [47]. With regard to an energetic particle link, a possible micro-physical pathway has been proposed by which weather systems and vorticity may be altered [61,75,76], which may be relevant to synoptic-scale weather.

Although the analyses of Huth *et al.* [20,65] differ to the one presented here methodologically, in many ways, the analyses are asking a similar question of the HBGWL dataset, thus it is interesting to contrast the results. While we observed some changes in circulation patterns that may be considered marginally significant, including during the winter months, we found a different pattern of change to those of Huth *et al.* [20,65]. Specifically, we find a weakly significant reduction in easterly weather types during solar maximum (similar to the positive NAO composite), not increases in the northeasterly and northwesterly flow as reported by Huth *et al.* [20]. We would argue that it is probable that the solar-related results identified in this work are not robust, a conclusion supported by the considerable uncertainty associated with the estimated significance of these samples and the fact that these results fall within the expected FDR. Consequently, we argue that this analysis has not shown strong evidence from the HBGWL data in support of a link between solar activity and WSD anomalies over Europe. Although, it is possible that the non-trivial complexity of analysing and interpreting these data, and the large uncertainty associated with their statistical evaluation may be the cause of the disagreement between our studies and the earlier positive results.

Highly significant changes in atmospheric flow were observed with peak phases of the NAO. It is possible that studies reporting positive associations between regional atmospheric flow and solar activity are suffering from chance coherence/interference with the NAO, similar to the noted impact of ENSO on global-scale solar–climate studies (e.g. [16,17]). Statistical difficulties with such studies could account for the reports of significant connections between the NAO and solar activity which have been found to break down and reverse sign (e.g. [67,77]). The authors have proposed hypotheses to account for such transient behaviour, relating to the interplay between geomagnetic activity and SCs of various amplitudes and phases. However, their results may simply result from the complexity of examining highly auto-correlated data. Such complexity has been recognized as a cause of significant uncertainty in the field of solar–climate studies. Pittock [14,78] summarized several non-trivial issues germane to this discussion.

First, weather and climate are highly variable over all timescales, yet only a small fraction of this variance could be reasonably ascribed to solar activity. This means that for solar–climate studies, the vast majority of the variance in relevant datasets may be considered noise, while only a small to potentially non-existent fraction of the variance could be linked to solar variability and thus considered signal. This imposes severe limits on the confidence of the conclusions which can be drawn from studies of statistical associations.

Second, as climatic data are spatially auto-correlated, increasing the number of observations globally does little to reduce uncertainty, meaning there is no substitute for long-duration datasets. As modern satellite-era datasets only cover around three SCs, few independent data points exist from which to evaluate solar–climate relationships.

In addition, it is highly problematic to disambiguate the forcing effects of internal variations in the climate system, such as volcanic eruptions or ENSO, which operate over timescales comparable to solar variability (e.g. [16–18]). This point is related to the second issue noted by Pittock, that long-term datasets are required to disambiguate forcing effects.

With regard to the positive result identified between southerly flow in boreal summertime and El Nino: associations between El Niño and temperature/precipitation anomalies in the Northern Hemisphere have been well established, and probably operate via teleconnections in both the troposphere and stratosphere [79]. However, the majority of the studies examining ENSO impacts on the climate of the Northern Hemisphere have focused on the boreal winter, as this is when El Nino tends to peak. While this does not preclude an ENSO influence on summer conditions in the Northern Hemisphere, it does mean that it is less understood.

We speculate that the rearrangement of the Walker circulation in association with ENSO may extend to the Atlantic basin, altering the sea surface temperature gradient and thereby influencing meridional energy flow. However, further studies are required to determine the precise mechanism which may explain our observations.

## 5. Conclusion

Using an epoch-superpositional (composite) methodology, with MC-based significance testing, we have examined subjectively classified HBGWL European weather type data for evidence of associations between the direction of atmospheric flow and peaks in various forcings. We found that the WSD shows a clear and highly significant response to peak phases of the NAO as expected: positive phases of the NAO were generally associated with statistically significant increases in the frequency of prevailing westerly flow, and reduced flow frequency from northerly–easterly directions, whereas negative NAO phases were found to be associated with inverse changes of approximately equal magnitude. The HBGWL data and the NAO index may be broadly thought of as representations of the same phenomena, thus we consider these results to confirm the utility of the HBGWL data, and also as a benchmark of a clear signal.

In contrast to the NAO-related responses, we failed to observe robustly significant changes in WSD frequency with peaks in the phases of the SC and ENSO, or peaks in stratospheric aerosols owing to volcanic activity. There was large uncertainty associated with these data, and several samples may be considered moderately significant, however, we consider that these results could be sufficiently explained by the FDR. We note that earlier studies have reported clearly significant positive associations between the HBGWL data and the SC, in conflict with our findings. However, we attribute this disagreement to difficulties in the analysis of these data.

There was one exception to these null results: we observed a highly significant increase in southerly weather types during boreal summer months in association with El Niño, of 2.6±0.8 days/month (*p*=1.9×10^−4^). Lagged analysis over a ±5 year period shows the signal to be centred on the year of peak signal, providing further support to the validity of this relationship. This is not the first time the HBGWL data have been reported to show positive associations to ENSO (e.g. [39]), and we consider this result to strongly support the existence of a teleconnection between El Niño and European weather.

## Data accessibility

Our data and code are available via Figshare Laken [24]. HBGWL data are from the Deutsche Wetterdienst (http://www.dwd.de). Stratospheric aerosol data from NASA (http://data.giss.nasa.gov/modelforce/strataer). MEI (ENSO) data from NOAA (http://www.esrl.noaa.gov/psd/enso/mei.ext/), credit Dr Klaus Wolter. NAO data from CRU (http://www.cru.uea.ac.uk/timo/datapages/naoi.htm/).
